# Integrative health care model for climacteric stage women: design of the intervention

**DOI:** 10.1186/1472-6874-11-6

**Published:** 2011-02-20

**Authors:** Svetlana V Doubova, Patricia Espinosa-Alarcón, Sergio Flores-Hernández, Claudia Infante, Ricardo Pérez-Cuevas

**Affiliations:** 1 Unidad de Investigación Epidemiológica y en Servicios de Salud, Centro Médico Nacional Siglo XXI. Instituto Mexicano del Seguro Social. México D.F., México; 2 Unidad de Investigación Educativa. Instituto Mexicano del Seguro Social. México D.F., México; 3 Coordinación de Investigación en Salud, Centro Médico Nacional Siglo XXI, Instituto Mexicano del Seguro Social. México D.F., México; 4 Facultad de Medicina. División de Estudios de Posgrado. Universidad Nacional Autónoma de México

## Abstract

**Background:**

Climacteric stage women experience significant biological, psychological and social changes. With demographic changes being observed in the growing number of climacteric stage women in Mexico, it is important to improve their knowledge about the climacteric stage and its potential associated problems, encourage their participation in screening programs, and promote the acquisition of healthy lifestyles.

At Mexican health care institutions the predominant health care model for climacteric stage women has a biomedical perspective. Medical doctors provide mostly curative services and have limited support from other health professionals. This study aims to design an integrative health care model (IHCM: bio-psycho-social, multidisciplinary and women-centered) applicable in primary care services aimed at climacteric stage women.

**Methods/Design:**

We present the design, inclusion criteria and detailed description of an IHCM. The IHCM consists of collaborative and coordinated provision of services by a health team, which is involves a family doctor, nurse, psychologist, and the woman herself. The health team promotes the empowerment of women through individual and group counseling on the climacteric stage and health related self-care. The intervention lasts three months followed by a three-month follow-up period to evaluate the effectiveness of the model. The effectiveness of the model will be evaluated through the following aspects: health-related quality of life (HR-QoL), empowerment, self-efficacy and knowledge regarding the climacteric stage and health-related self-care activities, use of screening services, and improvement in lifestyles (regular leisure time physical activity and healthy diet).

**Discussion:**

Participation in preventive activities should be encouraged among women in Mexico. Designing and evaluating the effectiveness of an integrative health care model for women at the climacteric stage, based on the empowerment approach and focus on health-related self-care to improve their HR-QoL is pertinent for current health conditions of this age group.

**Trial registration:**

The study is registered at the ClinicalTrials.gov (NCT01272115).

## Background

The climacteric stage is the transition from the reproductive to the non-reproductive period during a woman's life [1], and comprises 2 to 8 years before and after menopause [2], and coincides with a complex period in life due to biological, psychological and social changes.

Two circumstances motivated the design of this study. One is related to the unfelt and unsatisfied health needs of climacteric stage women; the other is related to the existing supply of health services in Mexico to the care of women at this stage.

### Health needs and existing supply of health services in Mexico for climacteric stage women

A need arises when one sees a difference between the situation considered as optimal and the actual observed situation; the difference to be reduced is then the need. In the health field, the felt need relates to the perception people have about their health problems or whatever they want to receive from the health services. Unlike a need that is not felt, there exists a need that people do not perceive, but it must be satisfied to maintain, restore or improve their health [3].

Increase in life expectancy has also caused an increase in the proportion of climacteric stage women with respect to the general population. Traditionally, climacteric stage women seek medical attention when they perceive health problems attributed to this stage such as severe climacteric symptoms, osteoporosis-related fractures, symptoms and/or complications of chronic diseases. Regarding the unfelt and unsatisfied need, most women do not perceive the need to prevent or detect such health problems early. These situations result in delayed health seeking-behavior for these problems, which in turn represent a significant burden for health care systems and for the society due to the high costs of health care, decreased quality of life and premature death [4-6].

In Mexico, a high proportion of women in the climacteric stage experience important biological, psychological and social changes; 50-70% report climacteric symptoms [7,8], 45.6% depression and other mood and sleep disorders [9], 31.1% suffer from hypertension, 16.7% suffer from diabetes [10,11]; the prevalence of hip fractures secondary to osteoporosis is 169 per 10,000 women-years [12], of breast cancer is 27.4 per 100,000 woman-years, and of cervical cancer is 19.2 per 100,000 woman-years [13]. This situation is worsened by women's overall limited knowledge about the potential health problems associated with the climacteric stage, and about the necessary preventive measures, indicated by their low participation in screening programs for chronic diseases and their unhealthy lifestyles (high proportion of physical inactivity and low consumption of fruits, vegetables and foods rich in calcium [8,11,14]), which impede a better overall health and quality of life.

Regarding the existing supply of health care for climacteric stage women (how the need is being met), Mexico has a fragmented health system, composed by Social Security institutions, the Ministry of Health (MoH) and private providers. The Mexican Institute of Social Security (IMSS) is a nation-wide social security institution that provides health and social services as well as economic benefits to formal sector workers and their families (approximately 48 million). While the MoH is central authority in charge of the policies and design of programs and while the provision of care is decentralized through the MoH in the 32 states, while granting each a certain autonomy, MoH provides health care to people without social security, mostly through the System for Social Protection of Health [15].

Despite the existence of preventive programs, most health care for climacteric women is based on a biomedical model in which a curative perspective predominates without support or with very limited support from other health professionals, where the relationship between health providers and patients is usually justified by authoritative professional knowledge. This model gives more importance to medical care for biological symptoms and minimizes the relevance of the psychogical and social changes that happen during the climacteric period.

### Interventions targeted at climacteric stage women

Most interventions for climacteric stage women have focused on informing/educating women about preventing certain diseases. Some studies have developed educational strategies to prevent osteoporosis [16] or to improve healthy diet [17], and have also aimed to improve physical activity in menopausal women, demonstrating the feasibility and effectiveness of moderate and rigorous physical activity, which in turn improves muscle strength, blood pressure, bone density and health-related quality of life (HR-QoL) [18-20]. Other studies have examined the impact of information about healthy behaviors, stress management and possible treatments during the climacteric stage; these studies have reported the favorable effect of education groups on the participants' knowledge, which is manifested by perceived ease in dealing with the changes of this stage, increase of healthy habits and increased sexual interest, when compared with a control group [21,22]. In climacteric women, as in the general population, interventions that incorporate cognitive-behavioral strategies, such as individual and group counseling to facilitate changes in lifestyle, have greater positive effect on vasomotor symptoms, psychological distress, self-efficacy, satisfaction with care and cardiovascular risk factors when compared to printed or verbal information [23-28].

### Integrative health care model and the empowerment approach

In the 1980 s, the biomedical model was strongly criticized in the middle of severe economic crises in many countries including Mexico. Alternative models that integrated biological, psychological and social areas gained momentum [29]. In 1994, M. Flint [30], highlighted the relationship among the biological, social and psychological factors that women face during menopause, highlighting the need for women to receive bio-psycho-social care at this stage.

The integrative health care model (IHCM) [31] includes a multidisciplinary approach based on a non-hierarchical combination of conventional medicine with complementary and alternative medicine, which promotes the harmonious continuity in decision-making and care that should be focused on the patient. The IHCM is based on a specific set of core values that include the goal of addressing health care in a holistic manner, and promoting personal health and wellbeing. The multidisciplinary approach of the IHCM is based on the collaborative participation of health team members, their mutual respect and a shared vision between them and the patients. This circumstance allows each provider and each patient to contribute their knowledge and particular skills to focus on providing health care to persons within individual care plans. In the experimental field in both developed and developing countries, attempts to implement the IHCM have been made. The study results showed that the IHCM allowed increased use of preventive services, improved health education and health outcomes [32,33]. The integrative care model is consistent with the empowerment approach. Empowerment is the social process of recognizing, promoting and enhancing the capacity of people to meet their needs, solve their problems, and mobilize the resources needed to take control of their lives [34]. The goal of patient empowerment is to build the patients' capacity for self-care, facilitate the process of obtaining experiences to identify problems (health needs), set goals related to their own health care, identify personal and social obstacles, develop solutions, consider the possible consequences of alternative solutions and make appropriate decisions for themselves [35-37]. The empowerment approach is linked to the theory of self-determination which supports the empowering of patients to be more independent in their self-care, and raising the possibility of long-lasting changes in health-related behaviors [38]. The counseling focused on the problems perceived and prioritized by the individual considers empowerment as an approach that also includes cultural aspects.

Interventions aimed to potentiate individual (or social) resources to prevent health damage or solve health problems implicitly adopt an empowerment approach to enable people to improve their quality of life by increasing the power to control their problems. For example, it has been shown that individual and group counseling on health-related self-care of patients with chronic diseases based on the empowerment principles, taking their cultural characteristics into account, achieved positive effects on HR-QoL, knowledge about the disease and related self-care, satisfaction with care and metabolic control compared with the control group (telephone counseling on metabolic control and traditional education) [39,40]. In addition, patients empowered in health-related self-care used fewer medical appointments, resulting in significant savings for health services [41].

### Conceptual model and objectives of the study

**Figure 1:**
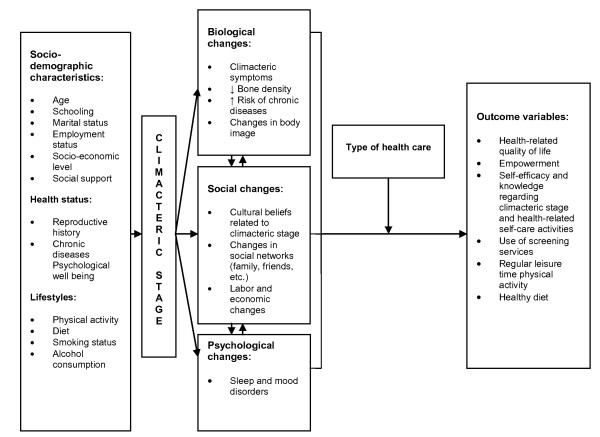
**Conceptual model of integrative health care for climacteric stage women**.

This framework sets the groundwork to address the health needs of climacteric stage women through an intervention that comprises, in an integral manner, their bio-psycho-social needs (Figure 1) and allows women to have greater control over the climacteric stage through acquiring knowledge, building on and utilizing individual strengths, making informed choices, setting personal goals, skills and attitudes necessary for better management of their environment and health-related self-care, which is called empowerment.

The characteristics of population, structure and processes of care within social security allows the implementation and testing of an integrative health care model (IHCM) for climacteric stage women (Table 1). The lessons learned from this first intervention that will be implemented in social security settings will provide further elements and evidence to eventually propose a study aimed at the population with social security in other developing countries. This work aims to develop an IHCM applicable in primary care services (family medicine clinics) for climacteric stage women. The effectiveness of the model will be evaluated through the following aspects: a) improved HR-QoL; b-d) increased empowerment, self-efficacy and knowledge regarding climacteric stage, and health-related self-care activities; e) increased use of screening services for breast cancer, cervical cancer, diabetes and hypertension; f-g) improved lifestyles: regular leisure time physical activity and healthy diet.

**Table 1:** Structure and population characteristics of health institutions in Mexico

	Mexican Institute of Social Security	Ministry of Health
Population	Formal sector workers and their families	People without formal employment contracts, or unemployed and the rural population
Organization	Centralize	Decentralize
Drug acquisition, laboratory and screening tests	Covered by the social insurance	Covered partially by the insurance + out of pocket payments
Medical doctors	1.4 per 1000 habitants	1.3 per 1000 habitants
Nurses	2.0 per 1000 habitants	1.7 per 1000 habitants

## Methods/Design

A field trial with one intervention and one comparison group, with ex ante and ex post measurements will be conducted in two IMSS primary care clinics in Mexico City. The clinics will be selected for convenience; each clinic must have more than 20 family doctors' offices and available space for the intervention (consulting room and an area for group counseling) and should have accepted to participate in the study; The intervention will be conducted in one clinic and the other clinic will serve as a comparison group, where the usual care for climacteric stage women will be observed.

The study population will consist of women affiliated with IMSS, between 45 and 59 years of age with a maximum of 5 years after menopause. The women should be users of the clinic, and should not have mental conditions that would prevent them from understanding the information or from taking independent decisions (dementia, mental retardation or psychosis), a physical disability that would impede them from participating (hearing loss, diseases affecting physical mobility as severe forms of rheumatoid arthritis), medical diagnosis of depression, diabetes mellitus, hypertension, renal or liver failure, and/or cancer, because patients with these diseases require specific care provided by several specialists. All women must agree to participate in the study through written informed consent.

The project was authorized by the National Research and Ethics Committee of the IMSS.

### Preparation of the intervention

In both intervention and control clinics the project will be presented to healthcare providers.

A healthcare team will implement the intervention comprised of family doctors, two nurses (one for each working shift) and a psychologist. The nurses and the psychologist will be selected from those with clinical experience in providing care to climacteric stage women; they will be previously trained by researchers and will work specifically in this project. The training will include theory and practice, and will last 60 hours based on a "training guide" that was developed *ex profeso*. The training topics include: 1) Health needs of climacteric stage women; 2) Risk factors for chronic diseases during the climacteric stage and self-care-related activities; 3) Health behaviors, self-efficacy, knowledge regarding climacteric stage and health-related self-care activities and empowerment; 4) Cognitive restructuring and problem solving, self-esteem and communication; 5) Healthy diet enriched with fruits, vegetables and milk products; 6) Regular leisure time with physical activity; 7) Individual counseling from the empowerment approach. Self-care plan: goal setting, action planning, monitoring and achievement of health goals; 8) Group counseling from the empowerment; 9) Integrative health care model for women at the climacteric stage.

All family doctors working in the intervention clinic will be invited to participate in the study. Family doctors, who agree to collaborate, will participate in a training course that consists in reviewing and discussing the proposed model, the roles and responsibilities of health providers involved in the care of climacteric stage women within IHCM. The researchers will deliver this course that will last two sessions of 60 minutes each.

### Description of the intervention

The IHCM with an empowerment approach will address the care that should be given collaboratively by the health team in cooperation with the active participation of women. The IHCM will be provided under the leadership of the nurse who will coordinate with the health team members, promote preventive care, and incorporate cognitive-behavioral strategies, such as individual and group counseling. The IHCM will be provided for three months to each woman, followed by a three-month follow-up. The empowerment of women will work to facilitate women's recognition of their actual or potential health problems, enable them to solve these problems by drawing up an action plan to improve or maintain their health, and develop their autonomous health-related self-care skills.

In the control group, women will continue the usual healthcare consisting of curative care provided by their family doctor and preventive care according to the IMSS prevention programs focused on this age group (chronic disease screening) and provided by nurses according to the women's demand.

### Recruitment of women and consultation with the family doctor

In both working shifts (morning and afternoon) family doctors will identify and evaluate those women who meet the inclusion criteria. Family doctors should provide preventive and curative healthcare services, according to womens' individual health needs, and send the candidates to the waiting room where they will be interviewed by study interviewers, who will explain the purpose and content of the study, and obtain the signed informed consent from those women who agree to participate. Once the participant is included in the study, the interviewer will perform the base-line interview then send the woman to the nurse of the study.

### Individual counseling by nurse

Nurses will provide monthly individual counseling to each woman based on the empowerment approach (the first visit being 40 to 60 minutes with subsequent counseling sessions of 20 to 30 minutes). The individual counseling will include the following contents (Table 2): 1) the nurse and the woman collaboratively evaluating the current status of the participant (physical, emotional, cognitive and so forth) within their social environment; 2) the nurse facilitating the women's recognition of her personal health risks and responsibilities for health-related self-care; 3) and 4) the nurse guiding the woman in setting her health goals and laying out a feasible action plan for her health-related self-care, thereby promoting the empowerment of each participant; and 5) the nurse and the woman collaboratively monitoring the progress of the action plan.

**Table 2:** Individual counseling for climacteric stage women

Steps	Content
1) Evaluation of the current status of women (physical, emotional, cognitive, etc.) within their social environment	• The nurse and the woman collaboratively assess the woman's health and health-related self-care practices (diet, leisure time physical activity, use of screening services).
	• The nurse provides relevant information on climacteric self-care: a) explains the definition of the climacteric stage and menopause and health problems the woman may experience during and after the climacteric stage; b) describe the factors (activities) that cause or protect against the occurrence of such problems; c) answers the woman's questions.
2) Facilitation of the woman's recognition of her personal health risks and responsibility for health-related self-care	• The nurse guides women on specific personal health-related values, beginning with the question: What health would you like to have in the next 10 years?
3 and 4) Guidance on the health-related goal setting and elaboration of a feasible action plan for women's self-care	• The nurse guides women to choose their personal health-related self-care goals.
	• The nurse guides women to choose strategies/actions to overcome barriers, using the problem-solving model.
	• The nurse facilitates the agreement of women to carry out the action plan.
	• The nurse and women jointly assess the level of womens' self-efficacy regarding the achievement of the agreed actions (on a scale of 0 to 10).
5) Monitoring of the progress of the action plan	The nurse and women jointly:
	• Evaluate progress in achieving the health-related goals and specific actions for development and maintenance of healthy behaviors.
	• Resolve problems related to barriers for implementing the action plan, translating such barriers in the "learned lessons."

Modified from Funnell MM, 2004 to counsel women at the climacteric stage.

The nurse will also schedule appointments for individual and group counseling and refer the woman to the psychologist and to the family doctor, who will provide comprehensive and continuous care consistent with the personal health needs of each participant.

### Individual counseling by the psychologist

Psychological counseling will be based on the Rational Emotive Behavioral Therapy and its central concept ABC [42]. According to this concept, emotional and behavioral consequences (C) are the result of beliefs (B) on activating events (A). The psychologist and the woman will collaboratively evaluate and restructure the woman's erroneous beliefs about the climacteric stage and her emotional resources, which refer to the ability to positively guide the emotions to achieve a better level of self-esteem and self-efficacy. The psychologist and the woman will define the number of consultations required in accordance to her individual needs and characteristics.

### Group counseling

All women of the intervention group will be invited to eight weekly, 90-minutes group sessions to facilitate and enhance their knowledge about menopause and their care, as well as to share experiences, concerns and motivations, with other women. Each group session will be coordinated by the nurse or psychologist of the study and will include five to ten women. The group sessions will focus on common educational needs. Both individual and group counseling will be supported by printed material developed *ex profeso*, which participants will be encouraged to keep as a source of information.

The main themes of group sessions will be: 1) the climacteric stage and the empowerment approach; 2) Emotional well-being, self-esteem and self-efficacy during menopause; 3) Factors that predispose chronic disease development during the climacteric stage and health-related self-care strategies; 4) Physical activity: barriers, benefits, type, frequency and recommended time. How to establish an individual action plan on physical activity and make it feasible (problem solving); 5) Healthy diet during the climacteric stage including increase of intake of fruits, vegetables and dairy products on a daily basis, as well as education about the barriers to and benefits of a healthy diet. How to establish an individual action plan toward a healthy diet and make it feasible; 6-8) Monitoring of the progress of women's action plans. Participants should attend to group sessions one to five, and to sessions six to eight can attend either as a group or individually.

### Data collection

Information on the study variables will be collected on three occasions: when the woman enters to the study (baseline), once intervention is complete (at three months) and after three more months of follow-up (six months).

### Study variables

The main outcome variables will be:

Health-related quality of life (HR-QoL), which will be measured with the revised version of Women's Health Questionnaire (WHQ23) [43]. This is a specific questionnaire for women in perimenopause, menopause and post menopause stages, that comprises six domains: anxiety/depression, well-being, somatic symptoms, memory/concentration, vasomotor symptoms and sleep problems. The WHQ23 also has two optional domains: sexual behavior and menstrual symptoms, which will apply only to women who are sexually active or who have menstrual periods. The highest value for each domain score is 100, which means high HR-QoL, and the lowest is 0, which means low HR-QoL.

Other outcome variables will be:

1) Empowerment of women in relation to their self-care measured by the empowerment scale, which was modified from the diabetes empowerment short scale [44] to be used in climacteric women. The maximum score of empowerment is 5, the minimum is 1.

2) Self-efficacy of women in relation to menopause and its care will be assessed with Perimenopausal Health Self-efficacy Scale (PHS-ES) which consists of 23 items [45]. The maximum score of PHS-ES is 207 (high self-efficacy) and the minimum is 23 (low self-efficacy).

3) Women's knowledge about the climacteric stage will be estimated with a questionnaire developed *ex profeso*. The questionnaire consists of 24 items that assess the following aspects: what is the climacteric stage, what are climacteric symptoms and health risks, and how can they be prevented during the climacteric stage. The minimum score on the scale corresponds to -24 points (without knowledge) and the maximum 24 points (full knowledge).

4) The use of screening services for: a) breast cancer by mammography in the last two years; b) cervical cancer by Pap-test in the last three years in women without a history of total hysterectomy; c) diabetes by measuring fasting plasma glucose in the last three years; d) hypertension: by measuring the systolic and diastolic blood pressure in the last year. The use of screening services will be estimated as the sum of the number of screening services that women used, divided by the total number of screening services recommended according to IMSS regulations and multiplied by 100.

5) Percentage of women who practice regular leisure time physical activity (PA), where regular was defined as moderate intensity if done for ≥150 minutes/week or vigorous intensity if done ≥75 minutes/week.

6) Percentage of women who practice a healthy diet, which included the daily consumption of fruits (3-4 servings), vegetables (4-5 servings), and dairy products (2-3 servings).

The covariates will be:

a) Women's general characteristics: Age, schooling, marital and employment status.

b) Smoking status and alcohol consumption. Smoking status will consider former and current smokers and will register the number of cigarettes actually smoked per day. Alcohol consumption will be classified as non-drinkers (never drink alcohol), occasional drinkers (drink rarely or less than once a week), moderate drinkers (from 1 to 14 drinks per week) and heavy drinkers (more than 14 drinks per week).

c) Nutritional status will be measured by body mass index (BMI) and classified into groups of normal weight (BMI of 18.5-24.9 kg/m^2^), overweight (BMI of 25.0 to 29.9 kg/m^2^), or obese (BMI ≥30.0 kg/m^2^).

d) Social support (SS) will be measured by applying the DUKE-UNC-11 questionnaire [46]. This questionnaire evaluates confidential SS (possibility of having people to communicate with) with a minimum score of 7 points (low confidential SS) and a maximum score of 35 points (high confidential SS); and affective SS (demonstration of love, affection, and empathy) with a minimum score of 4 (low affective SS) and a maximum score of 20 points (high affective SS).

e) Medical and reproductive history: Presence of chronic diseases, number of pregnancies and living children, and menopause (one year after the last menstrual period). Type of menopause will be classified as natural or surgical, age at onset of menopause; time elapsed since menopause, presence and type of climacteric symptoms, and number of visits with the family doctor or other health provider in the last three months. The severity of vasomotor symptoms and vaginal atrophy symptoms is classified using the criteria proposed by the Department of Health and Human Services Food and Drug Administration [47], which are based upon women's self-report, and define the symptoms as mild, moderate, or severe.

f) Satisfaction with care received at the FMC will be measured with the general question of how satisfied are you with the care you have received at the clinic? The possible answers are very satisfied, satisfied, neither satisfied nor unsatisfied, unsatisfied, and very unsatisfied.

g) Women's self-esteem will be measured with the Rosenberg Self-Esteem Scale (EAR) [48,49]. It is a one-domain instrument that consists of 10 questions. The highest score is 30 (high self-esteem) and the lowest is 0 (low self-esteem).

h) Adherence to MAIMC will be defined when the woman attends at least 60% of the planned individual and group counseling sessions.

### Sample size

The sample size for the primary outcome (HR-QoL) was estimated by using the formula to test change in the mean of two normally distributed samples in longitudinal studies [50]. An average increase of at least 10 points in one or more domains of WHQ23 in the intervention group compared with control group women was considered to be clinically relevant. The assumptions included: a mean HR-QoL score of 64.9 points (standard deviation of 23.4 points) in the domain of general well-being [51], α = 0.05 (for one-sided hypothesis) and β = 0.20. The number of women by group, assuming a drop-out rate of 20% will be 107.

We also estimated a sample size for regular leisure time physical activity considering that this is the outcome variable more difficult to achieve. It was assumed that only 17% of women in Mexico engaged in some type of regular leisure time physical activity [11] and a positive increase of at least 10% will be achieved after women participation in the IHCM. For this calculation, we used the formula to test the difference of proportions between two populations [50] with a = 0.05 (one side) and the power of 80%. The total number of women by group to include assuming a drop-out rate of 20% will be 207.

### Statistical analysis

The women characteristics of each intervention and comparison groups will be compared by using the Chi-square test to ascertain whether the groups were equivalent.

The effects of intervention for categorical outcome variables (regular leisure time physical activity, healthy diet) will be measured by comparing the baseline and post-intervention evaluation stages for differences in these variables using Chi-square test. Absolute percentage differences and corresponding 95% confidence intervals will be calculated.

Scores for the HR-QoL on the WHQ23 scale, the self-efficacy on the PHS-ES scale, the empowerment on the short form scale, knowledge about the climacteric stage, and menopause and self-care-related activities will be calculated according to the scoring algorithms. Measures of central tendency and distribution will be examined for these variables at the different measurement periods. The differences in the baseline and post-intervention stages for outcome variables will be compared within each group, and differences between the groups will be compared using the differences-in-differences (D-in-D) estimator [52]. The generalized linear model (GLM) will be used to assess changes in the main outcome variable controlling for baseline measures and other study variables as covariates. The GLM methods allow for modeling the correlation of the repeated observations on the same subjects [53].

The p value <0.05 will be considered as statistically significant. The analysis will be performed with the Stata 11.0 statistical software (Stata 11.0, Stata Corp; College Station, TX).

## Discussion

In primary care services, it is essential to provide a source of care that has continuity and coordination, and comprehensively covers the health needs of people. The constraints resulting from limited resources and restrictive organizational characteristics of institutionalized healthcare allows a limited range of changes, and these circumstances promote that innovative healthcare models should be carefully designed and evaluated to demonstrate healthcare service relevance and feasibility. Relevance, in this case, refers to meeting the health needs of climacteric stage women and to demonstrating its potential benefit. Feasibility means that the new model of care is applicable in the local context. An integrative health care model (bio-psycho-social, multidisciplinary and women-centered) with the empowerment approach may be a better alternative to ensure that women engage in their self-care and understand its importance to their present and future health and quality of life. The evaluation of the effectiveness of this research-based model can guide the feasibility of extending it into an operational model through a specific program.

The possible limitations of the study may include the following:

It was decided to perform the intervention in one clinic, taking another as a control to prevent women from both groups from communicating and transmitting information between them. However, the implementation of the study in two clinics may result in difference baseline characteristics of women in both groups. To overcome this limitation in the data analysis step, the regression methods will be used to adjust the results of the study for possible differences between groups.

Furthermore, the baseline evaluation can affect women in the control group resulting in them seeking counseling regarding the climacteric stage, attending screening programs, and changing their lifestyles.

The request for voluntary collaboration of family doctors and women could result in better doctors and women with healthier lifestyles agreeing to participate. Previous studies have reported that patients with unhealthy lifestyles are usually reluctant to participate. To confirm or disregard this limitation we will apply a short list of questions about the general characteristics and lifestyles to women who do not agree to participate in the study.

Compliance with the leisure time physical activity and healthy diet will be assessed through self-reporting of women unable to verify their actual practice, and will possibly overestimate the effect of the intervention regarding these variables.

Due to budgetary restrictions it is not possible to plan a long-term follow-up (12 months of study) of the IHCM impact.

Due to the financial and organizational feasibility, the study will be tested only in the population covered by IMSS, therefore, we can not say that this strategy would have the same results in women affiliated with other health institutions in Mexico, or women who receive healthcare from the Ministry of Health. However, we expect that the results of this study can be implemented in other institutions, with some necessary adaptations.

## Competing interests

The authors declare that they have no competing interests.

## Authors' contributions

SVD designed the study, educational materials and the training program, also wrote the paper. PEA contributed in the study design, development of the educational materials and the training program, also reviewed critically the manuscript. SFH contributed in the plan for the statistical analysis and reviewed critically the manuscript. CI contributed in the study design and reviewed critically the manuscript. RPC contributed in the study design and reviewed critically the manuscript. All authors read and approved the final manuscript.

## Pre-publication history

The pre-publication history for this paper can be accessed here:

http://www.biomedcentral.com/1472-6874/11/6/prepub
